# Effects of Superficial Scratching and Engineered Nanomaterials on Skin Gene Profiles and Microbiota in SKH-1 Mice

**DOI:** 10.3390/ijms242115629

**Published:** 2023-10-26

**Authors:** Kuunsäde Mäenpää, Marit Ilves, Lan Zhao, Harri Alenius, Hanna Sinkko, Piia Karisola

**Affiliations:** 1 Human Microbiome Research Program, Faculty of Medicine, University of Helsinki, 00014 Helsinki, Finland; kuunsade.maenpaa@helsinki.fi (K.M.); marit_ilves@hms.harvard.edu (M.I.); lan.zhao@helsinki.fi (L.Z.); harri.alenius@helsinki.fi (H.A.); hanna.sinkko@helsinki.fi (H.S.); 2 Institute of Environmental Medicine (IMM), Karolinska Institutet, 171 77 Stockholm, Sweden

**Keywords:** scratching, wound, mouse model, nanoparticles, metal oxides, RSEQ, inflammation, chemotaxis, microbiota

## Abstract

Scratching damages upper layers of the skin, breaks this first line of immune defence, and leads to inflammation response, which often also modifies the microbiota of the skin. Although the healing of incision wounds is well-described, there are fewer studies on superficial wounds. We used a simulated model of skin scratching to study changes in the host transcriptome, skin microbiota, and their relationship. Additionally, we examined the effect of nanosized ZnO, TiO_2_, and Ag on both intact and damaged skin. At 24 h after exposure, the number of neutrophils was increased, 396 genes were differentially expressed, and microbiota compositions changed between scratched and intact control skin. At 7 d, the skin was still colonised by gut-associated microbes, including *Lachnospiraceae*, present in the cage environment, while the transcriptomic responses decreased. To sum up, the nanomaterial exposures reduced the relative abundance of cutaneous microbes on healthy skin, but the effect of scratching was more significant for the transcriptome than the nanomaterial exposure both at 24 h and 7 d. We conclude that superficial skin scratching induces inflammatory cell accumulation and changes in gene expression especially at 24 h, while the changes in the microbiota last at least 7 days.

## 1. Introduction

The skin is a complex barrier against external environmental factors and is populated by diverse microbial communities [1]. The skin microbiota forms a barrier against colonisation by pathogenic bacteria and regulates cutaneous inflammatory and immune responses [2,3]. Disruptions in the skin–microbe interactions have been linked to skin disorders such as atopic dermatitis [4,5], although the exact mechanisms by which microbes influence disease development remain largely undefined.

Itching is a prevalent symptom in many skin diseases, and the subsequent scratching can significantly alter the skin’s structure and function [6]. Intense scratching damages both the epidermal and dermal layers, weakening the skin’s protective barrier, and triggering production of pro-inflammatory cytokines. Typical pruritic inflammatory skin disorders such as atopic dermatitis and contact dermatitis illustrate the consequences of persistent scratching. It has been convincingly shown that minimizing scratching alleviates the inflammatory response, potentially offering therapeutic benefits [7]. Additionally, such physical disruptions might also modify the skin microbiota, which is essential for maintaining skin health [8]. The role of microbiota in skin repair processes is dependent on the context, as the microbes can promote or even delay skin healing by, e.g., infecting wounds.

In modern applications, engineered nanomaterials (ENMs) are increasingly found in consumer products such as cosmetics, device coatings, and textiles [9]. Nanosized zinc oxide (nZnO) and titanium dioxide (nTiO_2_) are common components of sunscreens and various other cosmetic creams, which are intentionally and repeatedly spread on the skin. Sunscreens usually contain these materials in the size range of 30–150 nm [10,11,12]. Since certain nanomaterials possess antimicrobial properties [13,14,15], there is an interest in using ENMs in everyday and medical contexts against microbial infections. Nanosized silver (nAg) is generally utilised in the size range of 1–10 nm and is used in products such as antibacterial textiles and wound dressings, although in wound dressings, they can vary from 5 nm up to 150 nm [16,17]. Additionally, numerous surfaces, such as keyboards, may be coated with antimicrobial ENMs [11]. Therefore, there are various instances where the skin can be exposed to these ENMs. Although ENMs present distinct advantages, they also introduce potential health concerns, including cytotoxicity and unintended inflammatory responses [12,17,18]. Because of their potential to harm or benefit, it is essential to weigh the advantages of each ENM against its potential drawbacks and to decipher the molecular mechanisms behind their effects, aiming to minimise undesired outcomes.

Most research on skin aberration has utilised animal models with burn injuries [19] or wound incisions [20,21]. However, the effects of superficial scratching remain relatively unexplored. In our current study, we used hairless mice to assess the impact of controlled skin injuries caused by scratching, which mimics phenomena commonly observed in highly pruritic skin diseases. Additionally, we observed the effects of nZnO, nTiO_2_, and nAg on both intact and injured skin. We examined changes in the skin transcriptome and microbiota and their potential interactions. Our results provide new insights into host–microbiota relationships during skin inflammation and the impact of ENMs exposure on the skin microbiota.

## 2. Results

### 2.1. Scratching Recruited Neutrophils and Induced Immune Response Genes

**Figure 1:**
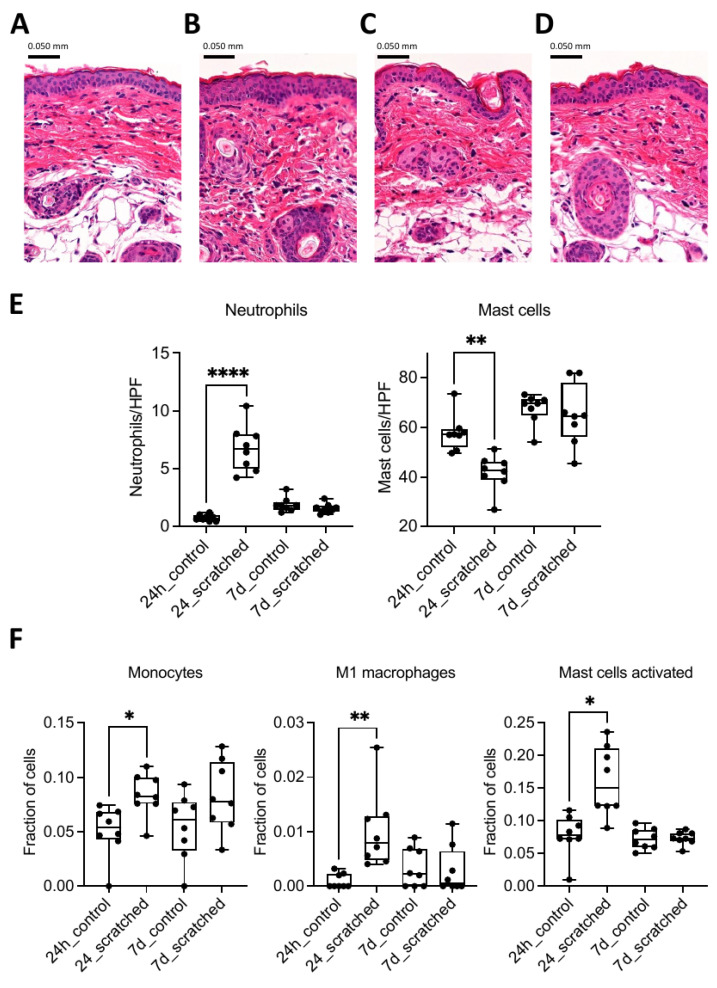
Haematoxylin and eosin (H&E)-stained mouse skin sections of (**A**) 24 h control group, (**B**) 24 h scratched group, (**C**) 7 d control group, and (**D**) 7 d scratched group. Pictures were taken with 200× magnification. (**E**) Neutrophils and mast cells were counted from H&E and toluidine blue-stained skin sections at 1000× and 400× magnification, respectively, under light microscope. (**F**) Deconvolution analysis of monocytes, M1 macrophages, and mast cells with the help of LM22 gene signature mouse orthologs. Stars indicate *p*-values: * < 0.05, ** < 0.01, **** < 0.0001.

**Figure 2:**
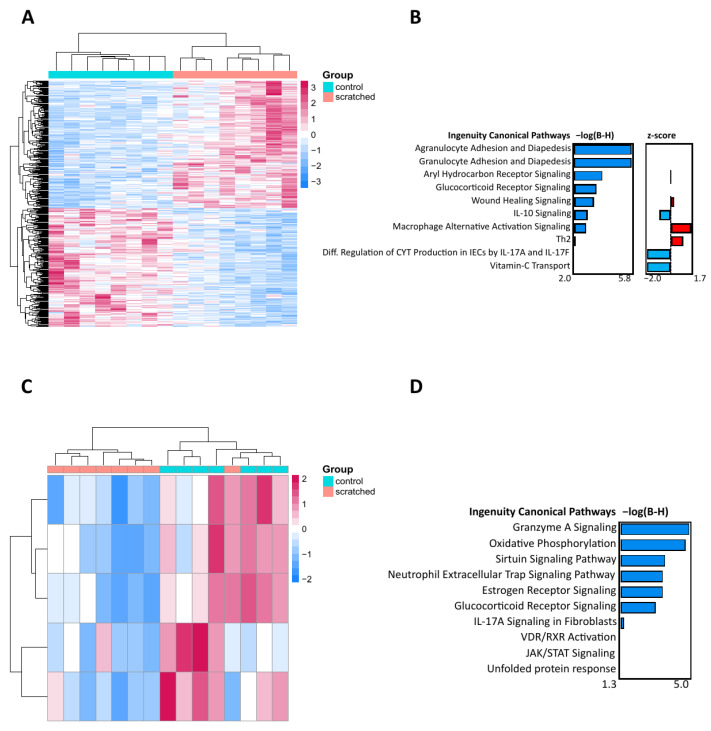
The effect of skin scratching at 24 h and 7 d on the transcriptome. (**A**) Heatmap of the 396 differentially expressed genes (DEGs) between the 24 h scratched group vs. 24 h control group formed two clear clusters. (**B**) The canonical pathways of 396 DEGs at 24 h highlight granulocyte and agranulocyte cell adhesion and diapedesis as identified by Qiagen IPA. (**C**) Heatmap of the five DEGs between the 7 d scratched group vs. 7 d control group, forming two clusters. (**D**) The canonical pathways of the five DEGs at 7 d show cell signalling and biosynthesis pathways as identified by Qiagen IPA. For heatmaps, Euclidean clustering distances were used for the complete clustering method and z scores are based on row scales. The DEGs with a *p* < 0.05 and a fold change smaller than |0.33| were included in the analysis. For the barplots, the DEGs are arranged via their Benjamini–Hochberg-corrected *p*-values.

To observe the effect of scratching on the skin, the scratched group was compared to the control group. In 24 h, the skin sections in the scratched group were slightly thicker compared to those in the control group (Figure 1A–D). The number of neutrophils was significantly higher, and the number of mast cells was significantly lower, but these differences were no longer observed at 7 days (Figure 1E). Based on deconvolution analysis, the number of monocytes and their differentiated subtype, M1 macrophages, as well as the number of activated mast cells, were elevated in the 24 h scratched group (Figure 1F). When comparing the transcriptomic responses from RNA sequencing in 24 h, 396 differentially expressed genes (DEGs) were identified between the scratched group and the control group (Figure 2A and Figure S1A,B). Qiagen IPA pathway analysis of DEGs revealed several pathways related to immune response, such as adhesion and diapedesis of granulocytes and agranulocytes, macrophage activation signalling, and wound healing (Figure 2B). After 7 days, there were only five DEGs (Cebpb, Ndufb5, Ndufb8, Ndufb9, and Rab5if) between the scratched and control groups, and they enriched to pathways on oxidative phosphorylation, granzyme A, sirtuin, and neutrophil extracellular trap-signalling pathways (Figure 2C,D and Figure S1B). The genes which were identified with PLS-DA as the most important between the groups enriched to biological processes such as intermediate filament organisation, supramolecular fibre organisation and epithelium development at both 24 h and 7 d timepoints (Figure S1C–F).

In addition to the effect of scratching, we studied the impact of nanosized zinc oxide (nZnO), silver (nAg), and titanium dioxide (nTiO_2_) exposure on intact and scratched skin (Figure S2). On day 7, there were 17 DEGs between nZnO and the control group, enriching pathways such as vitamin C transport and CLEAR signalling, and 10 DEGs between nAg and the control group, associated with cell signalling and biosynthesis pathways (Figure S3).

### 2.2. Skin Scratching Altered the Skin Microbial Community in 24 h

**Figure 3:**
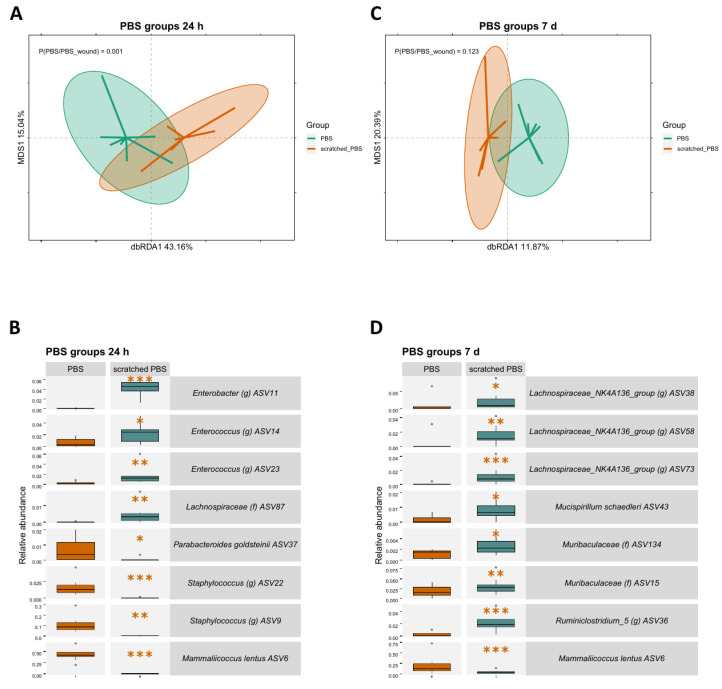
The effect of skin scratching at 24 h and 7 d on the skin microbiota. (**A**) A dbRDA analysis of microbial ASVs shows clear differentiation of the scratched and control groups at 24 h. Since the dbRDA analysis was performed for two groups, only one dbRDA axis is available for differentiation of the groups, and the MDS1 axis is used to create a two-dimensional figure. (**B**) Based on the generalised linear mixed model analysis, ASV6 *Mammaliicoccus lentus* and *Staphylococcus* ASVs are less abundant, while multiple gut-associated taxa are more abundant in the scratched 24 h group, based on the generalised linear mixed model analysis. (**C**) A dbRDA analysis of microbial ASVs at 7 d shows no difference between the groups. (**D**) Based on the generalised linear mixed model analysis, many gut-associated taxa are more abundant, and ASV6 *Mammaliicoccus lentus* is less abundant in the scratched group. Stars indicate *p*-values: * < 0.05, ** < 0.01, *** < 0.001. Ellipses on the top of symbols mark the 95% confidence interval, and the *p*-values of the ANOVA-like permutation test for dbRDA (permutations = 999) pairwise comparisons are included in each box.

Microbial changes induced by scratching were observed on the community level using dbRDA analysis and regarding individual amplicon sequence variants (ASV) using generalised linear mixed model analysis. The microbial communities were significantly different between the scratched group and control group at 24 h timepoint (*p* = 0.001, Figure 3A). In addition, distinct ASV abundances were observed between the groups. ASV6 representing *Mammaliicoccus lentus* (previous name *Staphylococcus lentus*), typical on animal skin, along with two ASVs representing *Staphylococcus*, had a lower abundance in the scratched than in the control group (Figure 3B). Conversely, ASVs associated with the gut, such as *Enterococcus*, had a higher abundance in the scratched group.

The microbial communities did not differ statistically between the scratched group and control group at the 7 d timepoint (Figure 3C). However, individual ASVs had differing abundances, with many gut-associated taxa, such as *Lachnospiraceae* and *Muribaculaceae*, being more abundant in the scratched group when compared to the control group (Figure 3D).

### 2.3. Bacterial Responses to Nanomaterials Were Different on Intact and Scratched Skin

**Figure 4:**
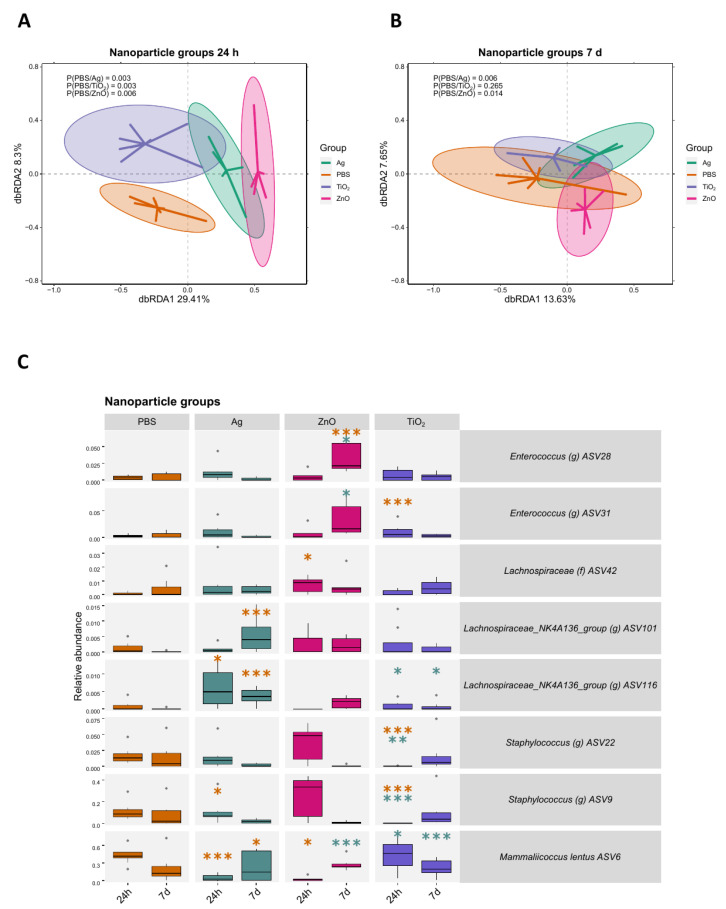
The effect of nAg, nZnO, or nTiO_2_ nanomaterial exposures on the intact skin microbiota 24 h and 7 days after the exposure. (**A**) A dbRDA analysis between the nAg, nZnO, or nTiO_2_ and control groups at 24 h shows microbial community differences between the nanomaterial groups and the control group. (**B**) A dbRDA analysis between the nAg, nZnO, or nTiO_2_ and control groups at 7 d no longer shows differences in the microbial communities between the groups. (**C**) ASV abundances changed depending on the material and timepoint. Results were based on the generalised linear mixed model analysis. For each nanomaterial group, the three ASVs which had the smallest FDR-corrected *p*-value were chosen for visualisation. Stars indicate *p*-values: * < 0.05, ** < 0.01, *** < 0.001. Orange stars indicate comparison to the control group, green stars indicate comparison to Ag group. Ellipses on the top of symbols mark the 95% confidence interval, and the *p*-values of the ANOVA-like permutation test for dbRDA (permutations = 999) pairwise comparisons are included in each box, n = 5–8.

The microbial community in each nanomaterial treatment group was significantly different from the control group at 24 h timepoint (Figure 4A). At the 7 d timepoint, the difference in the microbiota was significant between the control group and nAg and nZnO groups but no longer for nTiO_2_ group (Figure 4B). Compared to the control group, the change of ASV abundances after nanomaterial treatment was dependent on the nanomaterial and timepoint (Figure 4C). Notably, at the 24 h timepoint, ASV6 *Mammaliicoccus lentus* was less abundant in the nAg group (*p* < 0.001) and nZnO group (*p* < 0.05) but not in the nTiO_2_ group. At the 7 d timepoint, two *Lachnospiraceae* ASVs were more abundant in the nAg group (*p* < 0.001), and ASV28 *Enterococcus* was more abundant in the nZnO group (*p* < 0.001), when compared to control group.

**Figure 5:**
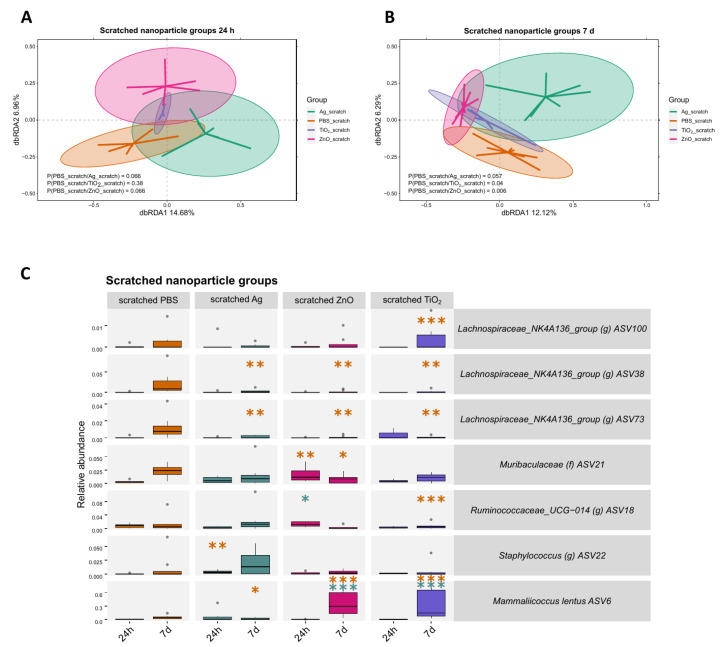
The effect of Ag, ZnO, or TiO_2_ nanomaterial exposures on the scratched skin microbiota 24 h and 7 days after the exposure. (**A**) A dbRDA analysis between the nAg, nZnO, or nTiO_2_ and control groups at 24 h does not show differences in the microbial community between the nanomaterial groups and the control group. (**B**) A dbRDA analysis between the nAg, nZnO, or nTiO_2_ and control groups at 7 d shows differences in the microbial community between nZnO and control group, as well as between nTiO_2_ and the control group. (**C**) ASV abundance changes were most notable at the 7 d timepoint based on the generalised linear mixed model analysis. For each nanomaterial group, the three ASVs which had the smallest FDR-corrected *p*-value were chosen for visualisation. Stars indicate *p*-values: * < 0.05, ** < 0.01, *** < 0.001. Orange stars indicate comparison to the control group, green stars indicate comparison to Ag group. Ellipses on the top of symbols mark the 95% confidence interval, and the *p*-values of the ANOVA-like permutation test for dbRDA (permutations = 999) pairwise comparisons are included in each box, n = 3–8.

In contrast to the changes in the microbiota observed between the nanomaterial treatment groups on intact skin, no differences in microbial communities were found between the nanomaterial and control group on scratched skin at 24 h timepoint (Figure 5A). However, the differences in microbiota emerged at the 7 d timepoint for nZnO and nTiO_2_ groups compared to the control group (Figure 5B). The most distinct variation in ASV abundances were observed at the 7 d timepoint, with two *Lachnospiraceae* ASVs having a lower abundance in all nanomaterial groups (*p* < 0.01, Figure 5C). In addition, some mice in the nTiO_2_ and nZnO groups, but not in the nAg group, had a higher abundance of *ASV6 Mammaliicoccus lentus* on their skin compared to the control group (*p* < 0.001).

### 2.4. Skin-Associated Taxa Correlated Negatively with Immune Response Induced by Scratching

**Figure 6:**
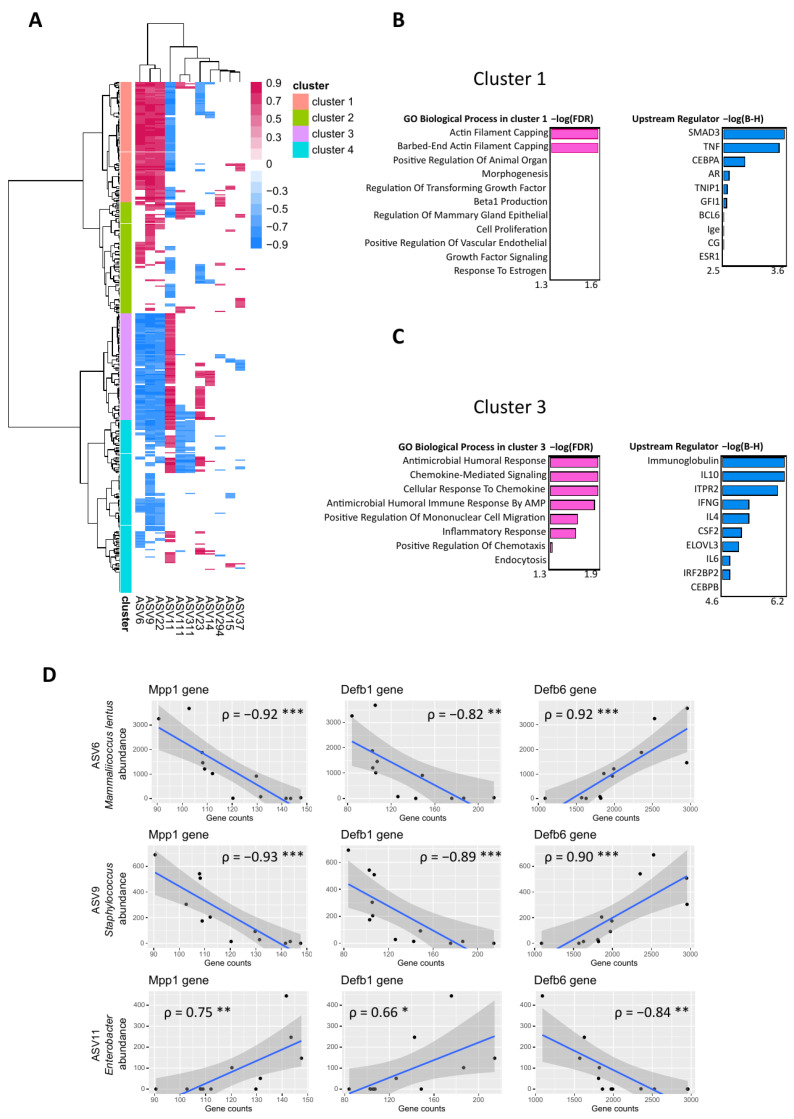
Spearman correlation analysis of DEGs and ASVs in the scratched and control group at 24 h. (**A**) Heatmap of Spearman correlation scores between 396 DEGs and 11/34 ASVs yielded four major clusters (1–4). ASVs whose absolute sum of columns was higher than 10 and correlations whose *p* < 0.05 were included in the figure. Euclidean distances were used to cluster the rows with the Ward D2 method. Biological processes and the upstream regulators of clusters 1 (**B**) and 3 (**C**) were identified by Qiagen IPA. (**D**) Spearman correlation analysis between individual genes and ASV6 *Mammaliicoccus lentus*, ASV9 *Staphylococcus*, and ASV11 *Enterobacter*. Outliers were excluded from ASV9 and ASV11. Stars indicate *p*-values: * < 0.05, ** < 0.01, *** < 0.001.

The relationship between gene expression in mouse skin and skin bacteria was further explored by performing a Spearman correlation analysis between DEGs and ASV abundances. The 396 DEGs between the scratched versus control groups were correlated with ASV abundances in these groups at 24 h timepoint. The clustering analysis of correlations grouped the ASVs into four clusters (Figure 6A). Cluster 1 consisted of multiple positive correlations between DEGs and three ASVs, namely, ASV6 *Mammaliicoccus lentus*, ASV9 *Staphylococcus*, and ASV22 *Staphylococcus.* Cluster 2 consisted of some positive correlations between these ASVs and the DEGs, cluster 3 consisted of multiple negative correlations of the three ASVs to the DEGs, and cluster 4 consisted of some negative correlations between these ASVs and DEGs. In contrast, ASV11 *Enterobacter* correlated positively with the DEGs when *M. lentus* and *Staphylococcus* ASVs correlated negatively in these clusters, and vice versa.

The clusters which had multiple positive or negative correlations between DEGs and ASVs, i.e., clusters 1 and 3, were analysed in Qiagen IPA and Enrichr to identify which molecules are involved in the regulation of these genes, upstream regulators, and what their biological processes are, respectively. In cluster 1, as in the cluster which included multiple positive correlations to *M. lentus* and *Staphylococcus* ASVs, upstream regulators such as SMAD3 and CEBPA and TNF were identified, and biological processes enriched to actin filament capping (Figure 6B). In cluster 3, i.e., the cluster with multiple negative correlations to *M. lentus* and *Staphylococcus* ASVs and upstream regulators such as immunoglobulin, IL10, and IFNG were identified, along with biological processes enriched to antimicrobial humoral response and chemokine signalling (Figure 6C). Further correlations were identified between ASVs and individual genes, namely, Defb6 from cluster 1, Mpp1, and Defb1 from cluster 3. ASV6 *Mammaliicoccus lentus* and ASV9 *Staphylococcus* correlated positively with the Defb6 gene and negatively with Mpp1 and Defb1 genes, while ASV11 *Enterobacter* correlated negatively with the Defb6 gene and positively with Mpp1 and Defb1 genes (Figure 6D).

## 3. Discussion

Superficial scratching is a key characteristic of pruritic skin conditions such as atopic dermatitis and may also occur in other instances such as skin irritation. However, its impact remains largely underexplored, especially concerning inflammatory profiles and cutaneous microbial communities. To address this gap, our current study used hairless mice to examine the effects of controlled skin injuries induced by scratching, thereby offering a closer approximation to skin conditions characterised by intense itching. Our study serves as a starting point, allowing future experiments to study these phenomena with skin disease models, whose gene expression and microbiota differ from models without disease. In addition to superficial scratching, we applied common nanomaterials—TiO_2_, ZnO, and Ag—to either intact or aberrated skin and assessed their effects on gene expression and the skin’s microbiota barrier.

The most dramatic changes in the scratched skin occurred at 24 h, both on the cellular and transcriptomic level. The number of neutrophils increased, and the number of mast cells decreased. With the help of deconvolution method, we showed that the numbers of monocytes, M1 macrophages, and activated mast cells were increased. An elevated number of recruited neutrophils and monocytes, which are differentiated in the tissue to inflammatory-type M1 macrophages, are typical for the inflammation stage of wound healing [22,23]. Inflammation induced degranulation of mast cells might explain the decreased number of mast cells at 24 h [24]. These results align with the enriched (a)granulocyte migration found in our pathway analyses of DEGs along with other immune response processes such as wound healing and downregulated IL-10 signalling. Other notable pathways at 24 h include aryl hydrocarbon and glucocorticoid receptor signalling, which are also shown to be important for skin regeneration and wound healing [25,26,27,28,29]. These results clearly indicate that scratching induces strong skin inflammation and cellular infiltration in 24 h.

In a week, the early infiltrated cells disappeared, and many other signs of inflammation were also resolved. At the 7 d timepoint, only five DEGs were identified between scratched and intact skin. The glucocorticoid receptor pathway was still enriched, along with energy metabolism pathways such as oxidative phosphorylation and sirtuin signalling pathways. Immune response pathways were still enriched, such as granzyme A and neutrophil extracellular trap signalling. The role of neutrophil extracellular trap related to healing of the injured skin is still unclear, as some studies conclude it enhances wound healing and some conclude it delays the process [30]. We additionally used the partial least squares-discriminant analysis (PLS-DA) to predict the most discriminative genes between scratched skins and control skins. Although there were some differences in individual genes between the 24 h and 7 d timepoints, the intermediate filament organisation and supramolecular fibre organisation were the most importantly varied processes. These processes were enriched by multiple keratin (Krt) genes, and they can either accelerate or slow down skin healing processes depending on the keratin type [31]. Altogether, these pathways at 7 d suggest an overlap of both inflammatory and proliferative stages in the healing of injured skin.

We also studied whether exposure to nanomaterials have any effect on the gene expression in the skin or its microbiota barrier. None of the particles induced any transcriptomic changes on the uninjured skin 24 h after the exposure. However, topical application of nZnO and nAg yielded minor numbers of DEGs in the skin transcriptome when compared to the control group at 7 d timepoint. The 10 DEGs after nAg treatment enriched to biosynthesis and cell cycle pathways. The enrichment of pathways related to vitamin C transport, CLEAR signalling, and senescence in the nZnO group suggests the involvement of cellular defence mechanisms, each with distinct roles: Vitamin C is an important antioxidant in the skin [32], CLEAR signalling is related to cellular clearance [33], and senescence is synonymous with aging. It is likely that these effects of nZnO or nAg on gene expression were indirect. There are hardly any studies exploring the effects of nTiO_2_ and nZnO on the skin transcriptome in vivo [34]. However, even repeated exposures over a long period of time have shown that the nZnO particles do not reach deeper layers of the skin [35,36], and only released ions from nAg can travel through the skin to the blood [37,38]. We additionally exposed the scratched skin to nanomaterials, but no genes were differentially expressed. This suggests that the effect of scratching had a more profound impact on transcriptomic responses compared to our administered dose of nanomaterials. Overall, these findings suggest that the effect of a single dose of nTiO_2_, nZnO, and nAg is minor on intact skin.

Even though the transcriptome changes were not significant on unbroken skin, microbial changes were evident. All nanomaterials altered the microbial community at 24 h, and the effect on individual microbial taxa depended on the nanomaterial. Most importantly, *Mammaliicoccus lentus* (ASV6) which was first isolated from animal skin [39,40], had a lower abundance after nAg and nZnO treatments. The impact of nTiO_2_ treatment on *M. lentus* was not significant. However, nTiO_2_ treatment lowered the abundance of *Staphylococcus* (ASV9 and ASV22), a commonly found microbial genus in skin [3,41]. At the 7 d timepoint, nAg and nZnO induced changes in microbial community compositions. Regarding individual taxa, nAg treatment resulted in a higher abundance of the family *Lachnospiraceae* (ASV101 and ASV116), which are commonly found in the mouse gut [42,43,44]. Additionally, nZnO exposure resulted in a higher abundance of *Enterococcus* (ASV28), also a gut-associated taxon. The nanomaterials utilised in our study are known to possess antimicrobial properties [14,15,45], likely contributing to the observed decrease native skin taxa. This created an opportunity for gut-derived taxa to colonise the skin. These bacteria were most likely transferred from the cage environment on to the skin, a phenomenon previously observed in our study [46]. We cannot exclude the possibility that bacteria from the cage environment were transferred to the skin as dead cells, as DNA-based sequencing cannot distinguish between different physiological statuses. Taken together, the antimicrobial properties of nanomaterials have the potential to reduce the relative abundance of native cutaneous microbes.

When it comes to the microbial changes in the scratched skin via nanomaterial exposure, it seemed that scratching was more significant than nanomaterial exposure, as there were no differences in the microbial communities at 24 h. The reliability of comparing microbial communities between the control group and the scratched nTiO_2_ group at the 24 h may be somewhat limited. This limitation arises from manufacturing-based contamination in our DNA extraction kit, which led to many samples failing our quality criteria for good-quality sequence analysis. However, this issue was successfully addressed by subjecting these samples to the careful decontamination process via the novel MicrobIEM decontamination tool [47]. Changes in microbial communities, as well as in individual taxa, were stronger at the 7 d timepoint. Interestingly, multiple *Lachnospiraceae* ASVs had a lower abundance after nanomaterial treatment compared to the control group. *Lachnospiraceae* and other gut-associated microbes were more abundant in the scratched control group, and nanomaterial treatment might have prevented colonisation by these microbes. Additionally, the abundance of *M. lentus* (ASV6) was high after nZnO and nTiO_2_ treatments, implying that *M. lentus* recovered faster due to the inhibited colonisation induced by nanomaterials. This was not the case with nAg treatment, after which the abundance of *M. lentus* was still low. It is possible that nAg had stronger antimicrobial properties, or the PVP coating on the nAg particles masked some of the antimicrobial properties.

Skin wounding and the resulting inflammation create a significant ecological change for the cutaneous microbiota [48]. In line with previous results, simulated scratching altered the microbial abundances in our model by changing the cutaneous environment. The change was disadvantageous for bacteria which are common on the skin, such as *M. lentus* (ASV6) and *Staphylococcus* (ASV9 and ASV22). This shift facilitated the colonisation of other bacteria from the cage environment; specifically, gut-associated ASVs in this case. Even though the entire microbial communities were not significantly altered after scratching at 7 d, individual ASVs had differing abundances compared to the control group. As was seen at 24 h, *M. lentus* was still less abundant in the scratched group at 7 d, while gut-associated taxa were more abundant. The relative abundance of *M. lentus* was very high at 24 h, but it has been described as a slow-growth microbe [39,40], which is likely why gut-derived taxa were still able to thrive after a week. In our previous study with tape-stripped mouse skin, the microbiota remained similar to the control after 29 days [49]. Since the skin is a harsh environment for the bacteria, reorganisation of the microbial community likely requires more time following significant skin aberration.

We further explored the relationship between host skin transcriptome and skin bacteria with respect to scratching at 24 h in our correlation analysis. *M. lentus* (ASV6) and *Staphylococcus* taxa (ASV9 and ASV22) correlated positively with genes whose biological processes enriched to cell organisation, organ morphogenesis, and cell proliferation. These pathways were predicted to be regulated by inflammatory TNF, which is produced by multiple cells such as macrophages and neutrophils during skin wound healing [22]. At the same time, the three skin ASVs correlated negatively with immune response and antimicrobial humoral response processes. *M. lentus* ASV6 and *Staphylococcus* ASV9 correlated negatively with Mpp1, which is associated to neutrophil chemotaxis [50], and Defb1, a gene for the antimicrobial peptide beta-defensin 1 [51]. In contrast, *Enterobacter* (ASV11) correlated positively with immune response processes. Most likely, the scratching-induced inflammation and the antimicrobial proteins such as Defb1 decreased the number of native cutaneous bacteria, such as *M. lentus* and *Staphylococcus*, creating a beneficial environment for *Enterobacter*, which is more often found in the gut. *Enterobacter* correlated negatively with beta-defensin 6 (Defb6), which has also been reported to have antimicrobial activity against another Gram-negative bacterium in the *Enterobacteriaceae* family, *Escherichia coli* [52]. Defb6 has also been reported to regulate other mouse skin commensals such as *Corynebacterium* [53]. Our results emphasise the importance of the native cutaneous microbiota in each study context, since the microbiota is highly influenced by the surrounding environment, such as the animal facility [41,44,46]. As the skin bacteria positively correlated with cell organisational processes, these results also suggest that native skin bacteria could play a significant role in skin wound healing and skin barrier functions.

## 4. Materials and Methods

### 4.1. Nanomaterials

Nanosized 30–40 nm TiO_2_ and nanosized 20 nm ZnO were purchased from Nanostructured and Amorphous Materials, Inc. (Houston, TX, USA), and nanosized 25 nm polyvinylpyrrolidone (PVP)-coated Ag were purchased from NanoComposix, Inc. (San Diego, CA, USA).

All particles were weighed in a sterile glass tube and were diluted with sterile 1X DPBS (Corning) into 50 mg/mL dispersions. Nanosized TiO_2_ and ZnO were sonicated for 20 min at 30 °C just before the treatment.

### 4.2. Mice

Female SKH-1 mice (n = 153) were obtained from Scanbur AB (Sollentuna, Sweden) and quarantined for one week. The mice were used in experiments at age of 7–8 weeks and were housed in transparent IVC plastic cages in groups of four, with each cage containing aspen chip bedding (Tapvei, Estonia). The mice were provided with tap water ad libitum and fed on a standard mouse chow diet. The animal facility had a 12 h dark–light cycle, a temperature of 20–21 °C, and relative humidity of 40–45%. The experiments were conducted in agreement with the European Convention for the Protection of Vertebrate Animals Used for Experimental and Other Scientific Purposes (Strasbourg 18 March 1986, adopted in Finland 31 May 1990). All experiments were approved by the State Provincial Office of Southern Finland (ESAVI/518/04.10.07/2017).

### 4.3. Animal Treatment Protocol

The mice were assigned into eight groups (8 per group) and two timepoints. Control mice in the “PBS” control group were treated with 10 μL of sterile DPBS (PBS) on their upper back, and the mice in the nanomaterial groups were treated with 10 μL of either nTiO_2_, nZnO, or nAg nanomaterial dispersion with a dose of 500 μg on the upper back. In the scratched groups, the upper back of the mice was first scratched with sterile metal brushes (brush hair length 2–3 mm, overall length 25 cm). The metal brushes were stroked six times away from the handler and then six times towards the handler, with a total of twelve strokes. Metal brushes were used to make the wounds superficial to simulate scratching. The PBS scratched group was then exposed to 10 μL of sterile PBS, and the scratched nanomaterial groups received a 500 μg in 10 μL of nanomaterials (nTiO_2_, nZnO, or nAg) on the scratches. After 24 h and 7 days, the mice were killed via isoflurane overdose. Swab samples were taken for microbiota analysis and stored at −80 °C, and biopsies were taken for histology and RNA analyses. Biopsy samples for RNA analysis were stabilised in RNALater solution (Invitrogen) for 24 h at +4 °C and thereafter stored at −80 °C until RNA extraction.

### 4.4. Histology

For histological analyses, a piece of the upper back biopsy was fixed in 10% buffered formalin for at least 24 h and embedded in paraffin. A total of 4 μm skin sections were cut and stained with haematoxylin and eosin (H&E) and toluidine blue to evaluate differences between treatment groups. Neutrophils were counted from the H&E-stained skin sections and mast cells from the toluidine blue stained sections. Both cell types were counted in the skin viable layer, the dermis, with a light microscope. Neutrophils were counted under 1000× total magnification and mast cells under 400× total magnification. A total of 5 high-power fields (HPF) were selected for each mouse, and the averages of the cell numbers from these fields were obtained to show the final number of each cell type per mouse. Neutrophils were recognised based on their structure, morphology and colour, i.e., neutrophils have a multilobed nucleus (between 2 and 5 lobes) and stain neutral pink. Mast cells were recognised based on their blue metachromatic granules, which were in the cytoplasm or near proximity of cell membrane in degranulated cells.

Skin sections were stained at the Tissue Preparation and Histochemisry Unit at the University of Helsinki. Images were generated using 3DHISTECH Pannoramic 250 FLASH III digital slide scanner at Genome Biology Unit supported by HiLIFE and the Faculty of Medicine, University of Helsinki, and Biocenter Finland.

### 4.5. RNA Extraction and Sequencing

Skin biopsies were transferred to 1 mL RL buffer (Norgen Biotek, Thorold, Canada) with a ¼ inch ceramic bead (MP Biomedicals, Santa Ana, CA, USA) and homogenised once or twice in the FastPrep machine (MP Biomedicals) at 6.0 m/s for 40 s. RNA was extracted using Total RNA Purification Plus Kit (Norgen Biotek) according to manufacturer’s instructions, except the samples were incubated for 2 min at RT before eluting to 50 μL of water. To remove traces of organic solvents, RNA was repurified using RNEasy Mini Cleanup kit (Qiagen, Venlo, Netherlands) according to the manufacturer’s instructions.

RNA was sequenced at the Functional Genomics Unit (FUGU) at the University of Helsinki, Finland. The libraries were sequenced on an Illumina NextSeq 500, with a read 1 custom primer producing read 1 of 21 bp and read 2 (paired end) of 62 bp. Raw sequence data were inspected using FastQC and multiQC to ensure the quality of sequencing results [54,55]. Subsequently, reads were filtered to remove reads shorter than 20 bp using Trimmomatic [56]. Reads passing the filter were then processed further using Drop-seq tools (v. 2.4.1) and the pipeline originally suggested in Drop-seq paper (v. 2.3.0) [57] and adapted to 3′ RNA-seq. Briefly, the raw, filtered read libraries were converted to sorted, unaligned BAM files using Picard tools (http://broadinstitute.github.io/picard, accessed on 23 May 2022). This was followed by tagging reads with sample specific barcodes and unique molecular identifiers (UMIs). Tagged reads were then trimmed for 5′ adapters and 3′ poly A tails. Alignment-ready reads were converted from BAM formatted files to fastq files that were used as an input for STAR aligner (v.2.7.6a) [58]. Alignments and gene annotations were based on Gencode mouse Release M28 (GRCm39) reference genome and gene annotation files with default STAR settings. Following the alignment, the uniquely aligned reads were sorted and merged with a previously unaligned tagged BAM file to regain barcodes and UMIs lost during the alignment step. Next, annotation tags were added to aligned and barcode tagged BAM files to complete the alignment process. Finally, Drop-seq tools were used to detect and correct systematic synthesis errors present in sample barcode sequences. Digital expression matrices were then created by counting the total number of unique UMI sequences (UMI sequences that differ only by a single base were merged together) for each gene. Gene count outputs from all sample pools were then compiled to form a single gene expression table for the whole dataset. The yielded data matrix of mapped and counted genes per sample was used as starting material for data analysis.

### 4.6. DNA Extraction and Sequencing

DNA extraction and PCR prior to sequencing were performed as previously described [46] except the swab samples were incubated in yeast cell lysis solution with 200 rpm shaking instead of 300 rpm. Extraction batch bias was mitigated as previously described, and control samples were obtained and treated as previously described [46].

Sequencing was performed at the Institute of Molecular Medicine Finland (FIMM, University of Helsinki) with paired-ends of 2 × 300 bp with Illumina MiSeq.

### 4.7. Pre-Processing of Raw 16S rRNA Gene Sequences

PCR primer sequences were removed using Cutadapt (v. 2.8, [59]) and initial quality analysis was conducted via MultiQC (v. 1.10.1, [55]). Subsequent analysis steps were performed in the R environment (v. 4.2.0 [60]) and with RStudio (v. 2023.03.1+446 [61]). The DADA2 package [62] was used to truncate the forward reads to 280 bp and reverse reads to 240 bp. Reads that had more than 3 expected errors were discarded during the filtering and trimming step. Sequence errors were removed with pseudo-pooling, the forward and reverse reads were merged, and chimeras were removed from the generated sequence table with default parameters to obtain amplicon sequence variants (ASVs) from the 16S rRNA gene sequences. These ASVs were assigned taxa and species by aligning them against the SILVA 132 database [63]. Non-bacterial ASVs and kingdom-level unidentified ASVs were removed. Contaminant ASVs were identified using MicrobIEM (v. 0.6 [47], https://github.com/LuiseRauer/MicrobIEM, accessed on 14 January 2022) with frequency mean ratio 0.1 and span threshold 0.1 for the first assigned negative control and frequency mean ratio 0.1 and span threshold 0.33 for the second assigned negative control. After decontamination, samples that had a library size between 1500 and 30,000 sequences were included in subsequent steps. Cumulative sum scaling [64] and count normalisation methods [65] were tested for the sequences, but they resulted in a high negative correlation rate between richness and library size (−0.80 for cumulative sum scaling and −0.83 for count normalisation method); therefore, only the relative abundance transformation was used as a way to equalise library sizes across samples.

### 4.8. Statistical Analyses

#### 4.8.1. Differential Expression Analysis of RNA-Sequencing Data

Initial filtering was performed with Perseus (v. 1.6.14.0) [66] so that genes which were observed in 6 out of 8 mice per group and had a higher count rate than 5 were included in subsequent steps. Differential gene expression was analysed using the R package DESeq2 [65]. Pairwise comparisons were fitted to a generalised linear mixed model with negative binomial distribution. Count normalisation was performed, the significant threshold for differentially expressed genes (DEGs) was set at fold change ≥ |0.33|, and Benjamini–Hochberg correction was performed on the *p*-values. Visualisation was performed using the R package pheatmap [67]. Pathways were identified using Qiagen IPA (QIAGEN Inc., Venlo, The Netherlands, https://digitalinsights.qiagen.com/IPA, accessed on 2 August 2023) [68]. The Venn diagram was visualised with Venny 2.1 [69].

#### 4.8.2. Leukocyte Deconvolution Analysis—CIBERSORT

The CIBERSORT [70] algorithm was used to estimate the leukocyte subset proportions of individual immune cells utilizing the mouse orthologs of “LM22” validated gene signature matrixes [71]. The estimations are based on 500 permutations. No significance filter has been applied to the estimated cell fractions to include all samples for further analysis.

#### 4.8.3. Partial Least-Squares Discriminant Analysis (PLS-DA)

Partial least-squares discriminant analysis (PLS-DA) was performed with the R package MetaboAnalystR (v. 4.0) [72,73]. The matrixes were normalised with quantile normalisation and scaled with mean-centre. PLS-DA cross-validation was performed to generate PLS importance analysis results. Biological processes were identified for the 30 most important genes using Enrichr [74,75,76].

#### 4.8.4. Evaluating Cage Effect on the Microbiota

Cage effect refers to a situation where the environment in individual cages of mice causes a cage-specific variation in microbial abundances between samples taken from mice. If not considered, the cage-specific variation may result in the false belief that the variation seen in the microbial communities is due to studied treatment. To identify which ASVs were strongly associated with cages, a generalised linear mixed model analysis was performed with negative binomial distribution using the R package lme4 [77]. Each ASV was treated as the response variable, the interaction of group and timepoint as explanatory variables, while cage and mice were included as random effects, and natural logarithm transformed library sizes were added as an offset. The ASVs whose random effect variance was higher than 1 were further analysed with distance-based redundancy analysis (dbRDA, see Section 4.8.5) to determine if the abundances of these ASVs were increased similarly in samples from the same cage. This resulted in low Bray–Curtis dissimilarity index values between these samples, which was not due to our experimental treatments. Based on this procedure, two ASVs were removed from the dataset.

#### 4.8.5. Distance-Based Redundancy Analysis (dbRDA)

Distance-based redundancy analysis (dbRDA) was performed with the R package vegan [78]. Relative abundances of ASVs were used to calculate Bray–Curtis dissimilarity indices between samples and input to a principal coordinates analysis (PcoA), followed by a redundancy analysis (RDA). In each subset, the group was used as an explanatory variable, and DNA extraction batches, library sizes, and richness were treated as confounders and partialled-out as conditioning variables. Richness was used as a confounding factor as, after the decontamination process, the number of sequences was substantially reduced in those samples that were strongly contaminated. This resulted in very high richness values that were not from biological origin but due to technical reasons and artificially associated with variations in the microbial communities in these samples. *p*-values were calculated with a, ANOVA-like permutation test for dbRDA pairwise comparisons using biodiversity R package [79] and then FDR corrected. The plots were visualised with the package ggplot2 [80].

#### 4.8.6. Linear Model

ASVs with a prevalence of over 30% in all samples were fitted in a generalised linear mixed model analysis. The data were subset pairwise according to timepoint and group. Using the lme4 package [77], the generalised linear mixed model was performed with negative binomial distribution with the control group of each pairwise subset as a baseline. Each ASV was treated as the response variable, group was treated as an explanatory variable, cage and mice were included as random effects, and natural logarithm-transformed library sizes were added as an offset. Visualisation was performed using ggordiplots [81] and ggplot2 packages [80].

#### 4.8.7. Correlation Analysis

Spearman Rho correlation tests [82] with FDR-corrected *p*-values were calculated between differentially expressed genes and ASV abundances and visualised using R packages pheatmap [67]. ASVs with a prevalence of over 50% were included in the correlation analysis. Rows were clustered in the heatmap via the Ward D2 method using Euclidean distances. The clustered dendrogram was clustered into four clusters with R internal “cutree” function and visualised with ggplot2 (Wickham 2016). Upstream regulators were identified using Qiagen IPA and biological processes with Enrichr (QIAGEN Inc., https://digitalinsights.qiagen.com/IPA, accessed on 2 August 2023) [68,74,75,76].

#### 4.8.8. Other Statistical Tests

Differences between numbers of neutrophils and mast cells were calculated via one-way ANOVA and Dunnett’s multiple comparison tests using GraphPad Prism. Differences between groups in deconvolution analysis were calculated via Kruskal–Wallis and Dunnett’s multiple comparison tests using GraphPad Prism.

## 5. Conclusions

Various skin diseases cause continuous itching, which leads to scratching and superficial wounds. To mimic this situation and follow the associated changes to the skin barrier and microbiota, we studied these effects on scratched skin with or without additional exposure to ENMs. It is important to note that our studies cover the effects on the skin transcriptome and microbiota in originally heathy conditions as we have not used a disease model. The majority of transcriptomic changes occurred in conjunction with an elevated number of neutrophils and M1 macrophages in the scratched skin after 24 h and played a role in the regulation of immune and inflammatory responses. These responses diminished in 7 days, and the skin started to regenerate. Considering the degree of transcriptional changes via superficial scratching, these results highlight the importance of wound healing systems in the skin. In terms of microbiota composition, it seems that skin scratching reduces cutaneous bacteria, and the resulting immune response does not allow native skin bacteria to return to homeostasis in a week. As for the nanomaterials, they had no impact on scratched skin transcriptome and induced only minor changes in the microbiome at the dosages used, suggesting the scratching was more significant on its own. However, nanomaterials caused differences in the intact skin microbiota that occurred especially after 7 d.

## Data Availability

The data presented in this study are available on request from the corresponding author.

## Supplementary Materials

The following supporting information can be downloaded at: https://www.mdpi.com/article/10.3390/ijms242115629/s1.

## References

[1] Lee A.Y. (2020). Molecular Mechanism of Epidermal Barrier Dysfunction as Primary Abnormalities. Int. J. Mol. Sci..

[2] Harris-Tryon T.A., Grice E.A. (2022). Microbiota and maintenance of skin barrier function. Science.

[3] Chen Y.E., Fischbach M.A., Belkaid Y. (2018). Skin microbiota-host interactions. Nature.

[4] Edslev S.M., Agner T., Andersen P.S. (2020). Skin Microbiome in Atopic Dermatitis. Acta Derm.-Venereol..

[5] Agrawal R., Woodfolk J.A. (2014). Skin barrier defects in atopic dermatitis. Curr. Allergy Asthma Rep..

[6] Yosipovitch G., Misery L., Proksch E., Metz M., Ständer S., Schmelz M. (2019). Skin Barrier Damage and Itch: Review of Mechanisms, Topical Management and Future Directions. Acta Derm.-Venereol..

[7] Weisshaar E., Bentz P., Haufe E., Heinrich L., Apfelbacher C., Heratizadeh A., Abraham S., Harder I., Kleinheinz A., Wollenberg A. (2023). Itching and treatments in atopic dermatitis (AD): Results from the German AD registry TREATgermany. Br. J. Dermatol..

[8] Canchy L., Kerob D., Demessant A., Amici J.M. (2023). Wound healing and microbiome, an unexpected relationship. J. Eur. Acad. Dermatol. Venereol..

[9] Khan I., Saeed K., Khan I. (2019). Nanoparticles: Properties, applications and toxicities. Arab. J. Chem..

[10] Schneider S.L., Lim H.W. (2019). A review of inorganic UV filters zinc oxide and titanium dioxide. Photodermatol. Photoimmunol. Photomed..

[11] Vance M.E., Kuiken T., Vejerano E.P., McGinnis S.P., Hochella M.F., Rejeski D., Hull M.S. (2015). Nanotechnology in the real world: Redeveloping the nanomaterial consumer products inventory. Beilstein J. Nanotechnol..

[12] Islam F., Shohag S., Uddin M.J., Islam M.R., Nafady M.H., Akter A., Mitra S., Roy A., Emran T.B., Cavalu S. (2022). Exploring the Journey of Zinc Oxide Nanoparticles (ZnO-NPs) toward Biomedical Applications. Materials.

[13] Bruna T., Maldonado-Bravo F., Jara P., Caro N. (2021). Silver Nanoparticles and Their Antibacterial Applications. Int. J. Mol. Sci..

[14] Lallo da Silva B., Abuçafy M.P., Berbel Manaia E., Oshiro Junior J.A., Chiari-Andréo B.G., Pietro R.C.R., Chiavacci L.A. (2019). Relationship between Structure and Antimicrobial Activity of Zinc Oxide Nanoparticles: An Overview. Int. J. Nanomed..

[15] Younis A.B., Haddad Y., Kosaristanova L., Smerkova K. (2023). Titanium dioxide nanoparticles: Recent progress in antimicrobial applications. Wiley Interdiscip. Rev. Nanomed. Nanobiotechnol..

[16] Krishnan P.D., Banas D., Durai R.D., Kabanov D., Hosnedlova B., Kepinska M., Fernandez C., Ruttkay-Nedecky B., Nguyen H.V., Farid A. (2020). Silver Nanomaterials for Wound Dressing Applications. Pharmaceutics.

[17] Ferdous Z., Nemmar A. (2020). Health Impact of Silver Nanoparticles: A Review of the Biodistribution and Toxicity Following Various Routes of Exposure. Int. J. Mol. Sci..

[18] Shi H., Magaye R., Castranova V., Zhao J. (2013). Titanium dioxide nanoparticles: A review of current toxicological data. Part. Fibre Toxicol..

[19] Valvis S.M., Waithman J., Wood F.M., Fear M.W., Fear V.S. (2015). The Immune Response to Skin Trauma Is Dependent on the Etiology of Injury in a Mouse Model of Burn and Excision. J. Investig. Dermatol..

[20] Saeed S., Martins-Green M. (2023). Animal models for the study of acute cutaneous wound healing. Wound Repair. Regen..

[21] Masson-Meyers D.S., Andrade T.A.M., Caetano G.F., Guimaraes F.R., Leite M.N., Leite S.N., Frade M.A.C. (2020). Experimental models and methods for cutaneous wound healing assessment. Int. J. Exp. Pathol..

[22] Cañedo-Dorantes L., Cañedo-Ayala M. (2019). Skin Acute Wound Healing: A Comprehensive Review. Int. J. Inflam..

[23] Krzyszczyk P., Schloss R., Palmer A., Berthiaume F. (2018). The Role of Macrophages in Acute and Chronic Wound Healing and Interventions to Promote Pro-wound Healing Phenotypes. Front. Physiol..

[24] Wilgus T.A., Wulff B.C. (2014). The Importance of Mast Cells in Dermal Scarring. Adv. Wound Care.

[25] Rico-Leo E.M., Lorenzo-Martín L.F., Román Á.C., Bustelo X.R., Merino J.M., Fernández-Salguero P.M. (2021). Aryl Hydrocarbon Receptor Controls Skin Homeostasis, Regeneration, and Hair Follicle Cycling by Adjusting Epidermal Stem Cell Function. Stem Cells.

[26] Carvajal-Gonzalez J.M., Roman A.C., Cerezo-Guisado M.I., Rico-Leo E.M., Martin-Partido G., Fernandez-Salguero P.M. (2009). Loss of dioxin-receptor expression accelerates wound healing in vivo by a mechanism involving TGFβ. J. Cell Sci..

[27] de Almeida T.F., de Castro Pires T., Monte-Alto-Costa A. (2016). Blockade of glucocorticoid receptors improves cutaneous wound healing in stressed mice. Exp. Biol. Med..

[28] Sanchis A., Alba L., Latorre V., Sevilla L.M., Pérez P. (2012). Keratinocyte-targeted overexpression of the glucocorticoid receptor delays cutaneous wound healing. PloS ONE.

[29] Tiganescu A., Hupe M., Uchida Y., Mauro T., Elias P.M., Holleran W.M. (2014). Increased glucocorticoid activation during mouse skin wound healing. J. Endocrinol..

[30] Zhu S., Yu Y., Ren Y., Xu L., Wang H., Ling X., Jin L., Hu Y., Zhang H., Miao C. (2021). The emerging roles of neutrophil extracellular traps in wound healing. Cell Death Dis..

[31] Cheng F., Eriksson J.E. (2017). Intermediate Filaments and the Regulation of Cell Motility during Regeneration and Wound Healing. Cold Spring Harb. Perspect. Biol..

[32] Pullar J.M., Carr A.C., Vissers M.C.M. (2017). The Roles of Vitamin C in Skin Health. Nutrients.

[33] Palmieri M., Impey S., Kang H., di Ronza A., Pelz C., Sardiello M., Ballabio A. (2011). Characterization of the CLEAR network reveals an integrated control of cellular clearance pathways. Hum. Mol. Genet..

[34] Wang S., Alenius H., El-Nezami H., Karisola P. (2022). A New Look at the Effects of Engineered ZnO and TiO_2_ Nanoparticles: Evidence from Transcriptomics Studies. Nanomaterials.

[35] Ilves M., Palomäki J., Vippola M., Lehto M., Savolainen K., Savinko T., Alenius H. (2014). Topically applied ZnO nanoparticles suppress allergen induced skin inflammation but induce vigorous IgE production in the atopic dermatitis mouse model. Part. Fibre Toxicol..

[36] Osmond-McLeod M.J., Oytam Y., Rowe A., Sobhanmanesh F., Greenoak G., Kirby J., McInnes E.F., McCall M.J. (2016). Long-term exposure to commercially available sunscreens containing nanoparticles of TiO_2_ and ZnO revealed no biological impact in a hairless mouse model. Part. Fibre Toxicol..

[37] Korani M., Rezayat S.M., Arbabi Bidgoli S. (2013). Sub-chronic Dermal Toxicity of Silver Nanoparticles in Guinea Pig: Special Emphasis to Heart, Bone and Kidney Toxicities. Iran. J. Pharm. Res..

[38] Tao H., Nagano K., Tasaki I., Zhang T.Q., Ishizaka T., Gao J.Q., Harada K., Hirata K., Tsujino H., Higashisaka K. (2020). Development and Evaluation of a System for the Semi-Quantitative Determination of the Physical Properties of Skin After Exposure to Silver Nanoparticles. Nanoscale Res. Lett..

[39] Kloos W.E., Schleifer K.H., Smith R.F. (1976). Characterization of *Staphylococcus sciuri* sp. nov. and Its Subspecies1. Int. J. Syst. Evol. Microbiol..

[40] Madhaiyan M., Wirth J.S., Saravanan V.S. (2020). Phylogenomic analyses of the *Staphylococcaceae* family suggest the reclassification of five species within the genus *Staphylococcus* as heterotypic synonyms, the promotion of five subspecies to novel species, the taxonomic reassignment of five *Staphylococcus* species to *Mammaliicoccus* gen. nov., and the formal assignment of *Nosocomiicoccus* to the family *Staphylococcaceae*. Int. J. Syst. Evol. Microbiol..

[41] Belheouane M., Vallier M.A.-O., Čepić A., Chung C.J., Ibrahim S., Baines J.A.-O. (2020). Assessing similarities and disparities in the skin microbiota between wild and laboratory populations of house mice. ISME J..

[42] Sun D., Bai R., Zhou W., Yao Z., Liu Y., Tang S., Ge X., Luo L., Luo C., Hu G.F. (2021). Angiogenin maintains gut microbe homeostasis by balancing α-Proteobacteria and Lachnospiraceae. Gut.

[43] Wang X., Wang Z., Cao J., Dong Y., Chen Y. (2023). Gut microbiota-derived metabolites mediate the neuroprotective effect of melatonin in cognitive impairment induced by sleep deprivation. Microbiome.

[44] Rosshart S.P., Herz J., Vassallo B.G., Hunter A., Wall M.K., Badger J.H., McCulloch J.A., Anastasakis D.G., Sarshad A.A., Leonardi I. (2019). Laboratory mice born to wild mice have natural microbiota and model human immune responses. Science.

[45] Sánchez-López E., Gomes D., Esteruelas G., Bonilla L., Lopez-Machado A.L., Galindo R., Cano A., Espina M., Ettcheto M., Camins A. (2020). Metal-Based Nanoparticles as Antimicrobial Agents: An Overview. Nanomaterials.

[46] Mäenpää K., Wang S., Ilves M., El-Nezami H., Alenius H., Sinkko H., Karisola P. (2022). Skin microbiota of oxazolone-induced contact hypersensitivity mouse model. PloS ONE.

[47] Hülpüsch C., Rauer L., Nussbaumer T., Schwierzeck V., Bhattacharyya M., Erhart V., Traidl-Hoffmann C., Reiger M., Neumann A.U. MicrobIEM—A User-Friendly Tool for Quality Control and Interactive Analysis of Microbiome Data. https://github.com/luiserauer/microbiem.

[48] Johnson T.R., Gómez B.I., McIntyre M.K., Dubick M.A., Christy R.J., Nicholson S.E., Burmeister D.M. (2018). The Cutaneous Microbiome and Wounds: New Molecular Targets to Promote Wound Healing. Int. J. Mol. Sci..

[49] Karisola P., Suomalainen A., Fortino V., Ottman N., Vendelin J., Wolff H.J., Ruokolainen L., Greco D., Fyhrquist N., Alenius H. (2019). Tape-stripping alters the microbe-host correlations in mouse skin. Allergy.

[50] Quinn B.J., Welch E.J., Kim A.C., Lokuta M.A., Huttenlocher A., Khan A.A., Kuchay S.M., Chishti A.H. (2009). Erythrocyte scaffolding protein p55/MPP1 functions as an essential regulator of neutrophil polarity. Proc. Natl. Acad. Sci. USA.

[51] Morrison G., Kilanowski F., Davidson D., Dorin J. (2002). Characterization of the mouse beta defensin 1, Defb1, mutant mouse model. Infect. Immun..

[52] Yamaguchi Y., Fukuhara S., Nagase T., Tomita T., Hitomi S., Kimura S., Kurihara H., Ouchi Y. (2001). A novel mouse beta-defensin, mBD-6, predominantly expressed in skeletal muscle. J. Biol. Chem..

[53] Sakamoto K., Jin S.P., Goel S., Jo J.H., Voisin B., Kim D., Nadella V., Liang H., Kobayashi T., Huang X. (2021). Disruption of the endopeptidase ADAM10-Notch signaling axis leads to skin dysbiosis and innate lymphoid cell-mediated hair follicle destruction. Immunity.

[54] Andrews S. FastQC: A Quality Control Tool for High Throughput Sequence Data. http://www.bioinformatics.babraham.ac.uk/projects/fastqc.

[55] Ewels P., Magnusson M., Lundin S., Käller M. (2016). MultiQC: Summarize analysis results for multiple tools and samples in a single report. Bioinformatics.

[56] Bolger A.M., Lohse M., Usadel B. (2014). Trimmomatic: A flexible trimmer for Illumina sequence data. Bioinformatics.

[57] Macosko E.Z., Basu A., Satija R., Nemesh J., Shekhar K., Goldman M., Tirosh I., Bialas A.R., Kamitaki N., Martersteck E.M. (2015). Highly Parallel Genome-wide Expression Profiling of Individual Cells Using Nanoliter Droplets. Cell.

[58] Dobin A., Davis C.A., Schlesinger F., Drenkow J., Zaleski C., Jha S., Batut P., Chaisson M., Gingeras T.R. (2013). STAR: Ultrafast universal RNA-seq aligner. Bioinformatics.

[59] Martin M. (2011). Cutadapt removes adapter sequences from high-throughput sequencing reads. EMBnet.Journal.

[60] R Core Team R: A Language and Environment for Statistical Computing. https://www.r-project.org/.

[61] RStudio Team Rstudio: Integrated Development for R. http://www.rstudio.com/.

[62] Callahan B.J., McMurdie P.J., Rosen M.J., Han A.W., Johnson A.J.A., Holmes S.P. (2016). DADA2: High-resolution sample inference from Illumina amplicon data. Nat. Methods.

[63] Quast C., Pruesse E., Yilmaz P., Gerken J., Schweer T., Yarza P., Peplies J., Glöckner F.O. (2012). The SILVA ribosomal RNA gene database project: Improved data processing and web-based tools. Nucleic Acids Res..

[64] Paulson J.N., Stine O.C., Bravo H.C., Pop M. (2013). Differential abundance analysis for microbial marker-gene surveys. Nat. Methods.

[65] Love M.I., Huber W., Anders S. (2014). Moderated estimation of fold change and dispersion for RNA-seq data with DESeq2. Genome Biol..

[66] Tyanova S., Temu T., Sinitcyn P., Carlson A., Hein M.Y., Geiger T., Mann M., Cox J. (2016). The Perseus computational platform for comprehensive analysis of (prote)omics data. Nat. Methods.

[67] Kolde R. (2012). Pheatmap: Pretty heatmaps. R Package Version.

[68] Krämer A., Green J., Pollard J., Tugendreich S. (2014). Causal analysis approaches in Ingenuity Pathway Analysis. Bioinformatics.

[69] Oliveros J.C., Venny (2007). An Interactive Tool for Comparing Lists with Venn’s Diagrams. https://bioinfogp.cnb.csic.es/tools/venny/.

[70] Newman A.M., Steen C.B., Liu C.L., Gentles A.J., Chaudhuri A.A., Scherer F., Khodadoust M.S., Esfahani M.S., Luca B.A., Steiner D. (2019). Determining cell type abundance and expression from bulk tissues with digital cytometry. Nat. Biotechnol..

[71] Newman A.M., Liu C.L., Green M.R., Gentles A.J., Feng W., Xu Y., Hoang C.D., Diehn M., Alizadeh A.A. (2015). Robust enumeration of cell subsets from tissue expression profiles. Nat. Methods.

[72] Chong J., Soufan O., Li C., Caraus I., Li S., Bourque G., Wishart D.S., Xia J. (2018). MetaboAnalyst 4.0: Towards more transparent and integrative metabolomics analysis. Nucleic Acids Res..

[73] Chong J., Xia J. (2018). MetaboAnalystR: An R package for flexible and reproducible analysis of metabolomics data. Bioinformatics.

[74] Chen E.Y., Tan C.M., Kou Y., Duan Q., Wang Z., Meirelles G.V., Clark N.R., Ma’ayan A. (2013). Enrichr: Interactive and collaborative HTML5 gene list enrichment analysis tool. BMC Bioinform..

[75] Kuleshov M.V., Jones M.R., Rouillard A.D., Fernandez N.F., Duan Q., Wang Z., Koplev S., Jenkins S.L., Jagodnik K.M., Lachmann A. (2016). Enrichr: A comprehensive gene set enrichment analysis web server 2016 update. Nucleic Acids Res..

[76] Xie Z., Bailey A., Kuleshov M.V., Clarke D.J.B., Evangelista J.E., Jenkins S.L., Lachmann A., Wojciechowicz M.L., Kropiwnicki E., Jagodnik K.M. (2021). Gene Set Knowledge Discovery with Enrichr. Curr. Protoc..

[77] Bates D., Mächler M., Bolker B., Walker S. (2015). Fitting linear mixed-effects models using lme4. J. Stat. Softw..

[78] Oksanen J., Blanchet F.G., Friendly M., Roeland K., Legendre P., McGlinn D., Minchin P.R., O’Hara R.B., Simpson G.L., Solymos P. Vegan: Community Ecology Package. https://cran.r-project.org/package=vegan.

[79] Kindt R., Coe R. (2005). Tree Diversity Analysis. A Manual and Software for Common Statistical Methods for Ecological and Biodiversity Studies.

[80] Wickham H. (2016). ggplot2: Elegant Graphics for Data Analysis.

[81] Quensen J. ggordiplots: Make ggplot Versions of Vegan’s Ordiplots. http://github.com/jfq3/ggordiplots.

[82] Harrell F.E. Hmisc: Harrell Miscellaneous. https://cran.r-project.org/web/packages/.

