# Supplementary material for: Effects of Superficial Scratching and Engineered Nanomaterials on Skin Gene Profiles and Microbiota in SKH-1 Mice
Source: Int J Mol Sci. 2023 Oct 26;24(21):15629. doi: 10.3390/ijms242115629 (PMC10649582; doi:10.3390/ijms242115629)
Supplement: Supplementary file 1 [file ijms-24-15629-s001.zip › FIGS1.pdf]

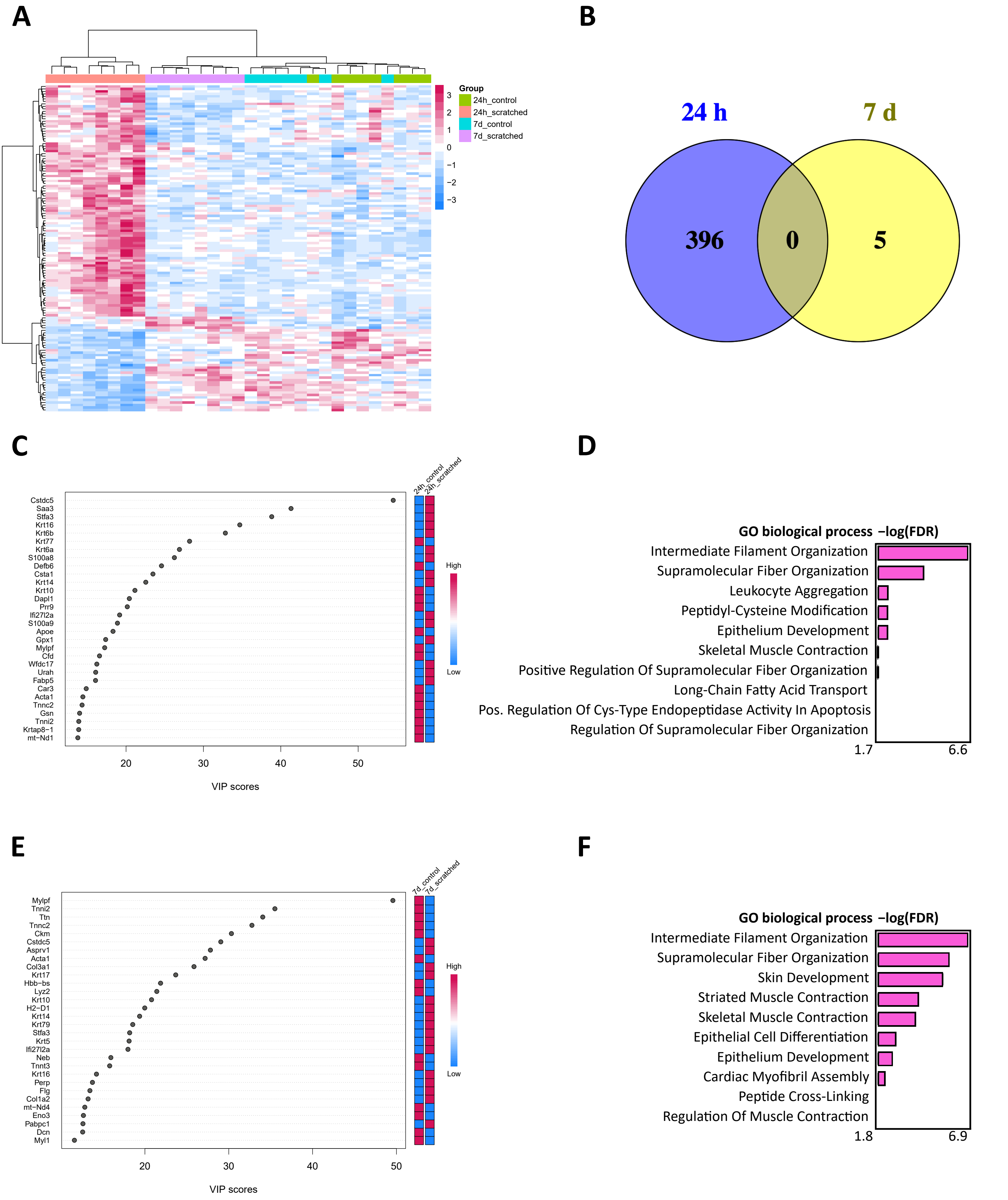

Figure S1. Most discriminatory and differentially expressed genes and biological processes in the scratched groups. (**A**) Heatmap of the top 150 most discrimantory genes, based on ANOVA. (**B**) Venn diagram of the 401 DEGs at 24 h and 7 d timepoints. PLS-DA analysis shows the top 30 genes based on their VIP scores at 24 h and their GO biological processes at 24 h (**C-D**) and at 7 d (**E-F**). The associated heatmaps in graphs C-D indicate upregulated (red) or downregulated (blue) expression of the genes in scratched versus control skins. For the heatmap, Euclidean clustering distances were used with the Ward D2 method.
