# Supplementary material for: Effects of Superficial Scratching and Engineered Nanomaterials on Skin Gene Profiles and Microbiota in SKH-1 Mice
Source: Int J Mol Sci. 2023 Oct 26;24(21):15629. doi: 10.3390/ijms242115629 (PMC10649582; doi:10.3390/ijms242115629)
Supplement: Supplementary file 1 [file ijms-24-15629-s001.zip › FIGS3.pdf]

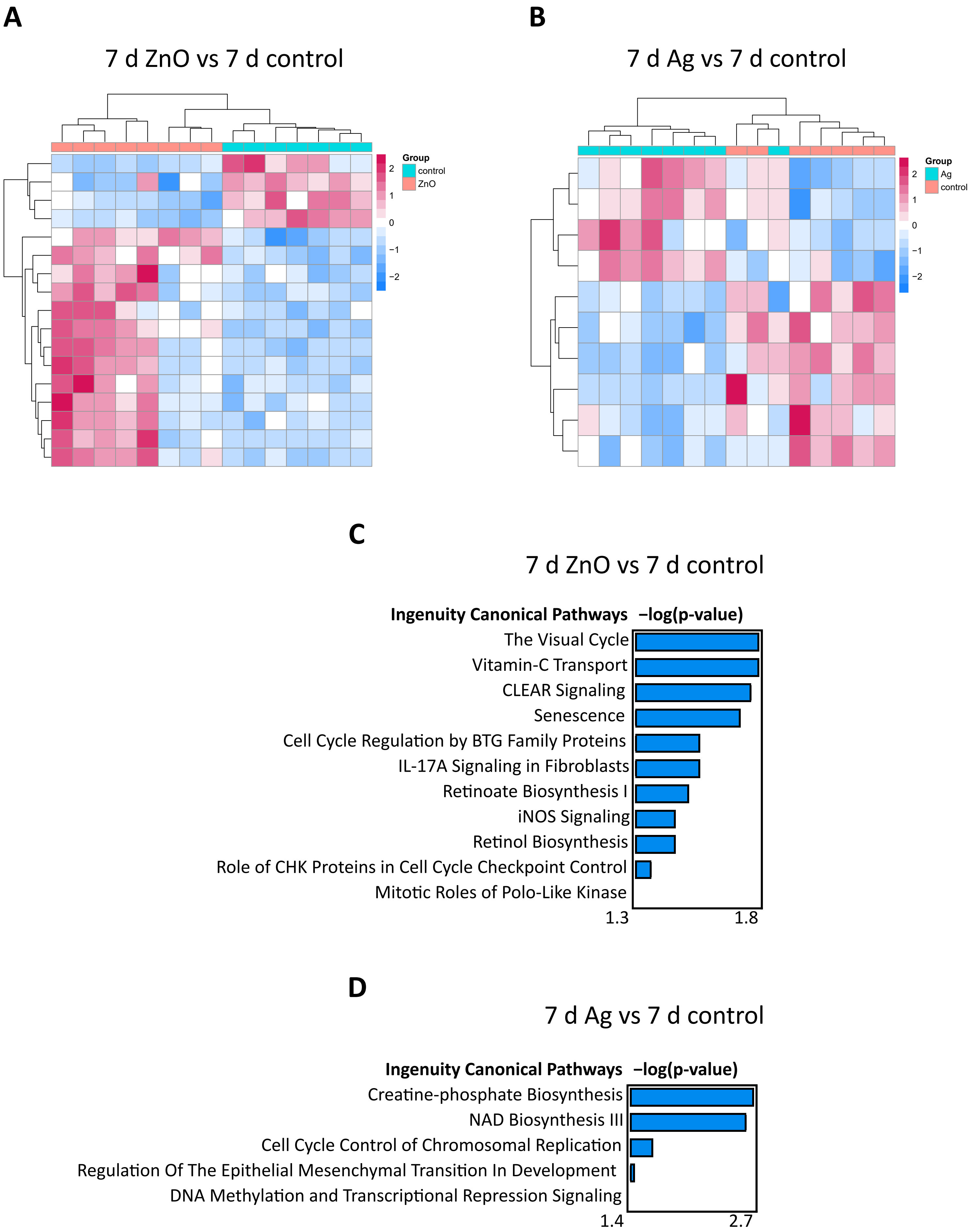

Figure S3. DEGs between nanomaterial treated and control groups at 7 d. (**A**) A heatmap of the 17 DEGs between 7 d nZnO group and 7 d control group. (**B**) A heatmap of the 10 DEGs between 7 d nAg group and 7 d control group. (**C**) DEGs between 7 d nZnO and 7 d control group enriched to cell signalling pathways. (**D**) DEGs between 7 d nAg and 7 d control group include biosynthesis pathways. DEGs are arranged by their p values. Canonical pathway analyses were performed with Qiagen IPA.
