# Supplementary figures and images for: Effects of Superficial Scratching and Engineered Nanomaterials on Skin Gene Profiles and Microbiota in SKH-1 Mice
Source: Int J Mol Sci. 2023 Oct 26;24(21):15629. doi: 10.3390/ijms242115629 (PMC10649582; doi:10.3390/ijms242115629)

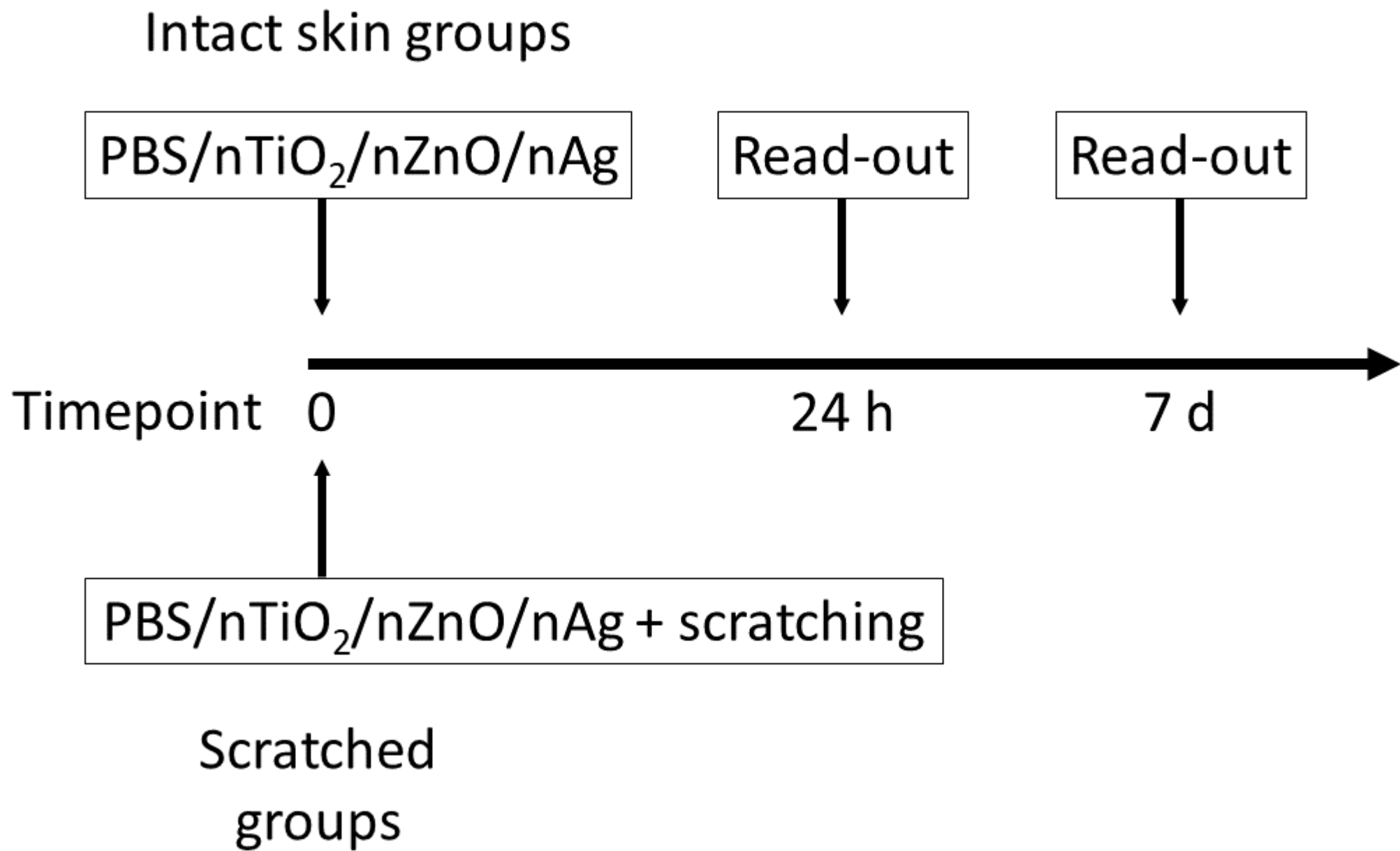

**Figure S2.** Schematic of the *in vivo* experiment.

Supplement: Supplementary file 1 [file ijms-24-15629-s001.zip › FIGS2.pdf]
